# Effect of continuous glucose monitoring compared with self-monitoring of blood glucose in gestational diabetes patients with HbA1c<6%: a randomized controlled trial

**DOI:** 10.3389/fendo.2023.1174239

**Published:** 2023-04-19

**Authors:** Mengyu Lai, Jianrong Weng, Jiaying Yang, Yujia Gong, Fang Fang, Na Li, Mei Kang, Xianming Xu, Yufan Wang

**Affiliations:** ^1^ Department of Endocrinology and Metabolism, Shanghai General Hospital, Shanghai Jiao Tong University School of Medicine, Shanghai, China; ^2^ Department of Obstetrics and Gynecology, Shanghai General Hospital, Shanghai Jiao Tong University School of Medicine, Shanghai, China; ^3^ Clinical Research Center, Shanghai General Hospital, Shanghai Jiao Tong University School of Medicine, Shanghai, China

**Keywords:** gestational diabetes mellitus, continuous glucose monitoring, self-monitoring of blood glucose, gestational weight gain, cost

## Abstract

**Objective:**

This study evaluated the effect of continuous glucose monitoring (CGM) versus self-monitored blood glucose (SMGB) in gestational diabetes mellitus (GDM) with hemoglobin A1c (HbA1c) <6%.

**Methods:**

From January 2019 to February 2021, 154 GDM patients with HbA1c<6% at 24–28 gestational weeks were recruited and assigned randomly to either SMBG only or CGM in addition to SMBG, with 77 participants in each group. CGM was used in combination with fingertip blood glucose monitoring every four weeks until antepartum in the CGM group, while in the SMBG group, fingertip blood glucose monitoring was applied. The CGM metrics were evaluated after 8 weeks, HbA1c levels before delivery, gestational weight gain (GWG), adverse pregnancy outcomes and CGM medical costs were compared between the two groups.

**Results:**

Compared with patients in the SMBG group, the CGM group patients had similar times in range (TIRs) after 8 weeks (100.00% (93.75-100.00%) versus 99.14% (90.97-100.00%), *p*=0.183) and HbA1c levels before delivery (5.31 ± 0.06% versus 5.35 ± 0.06%, *p*=0.599). The proportion with GWG within recommendations was higher in the CGM group (59.7% versus 40.3%, *p*=0.046), and the newborn birth weight was lower (3123.79 ± 369.58 g versus 3291.56 ± 386.59 g, *p*=0.015). There were no significant differences in prenatal or obstetric outcomes, e.g., cesarean delivery rate, hypertensive disorders, preterm births, macrosomia, hyperbilirubinemia, neonatal hypoglycemia, respiratory distress, and neonatal intensive care unit admission >24 h, between the two groups. Considering glucose monitoring, SMBG group patients showed a lower cost than CGM group patients.

**Conclusions:**

For GDM patients with HbA1c<6%, regular SMBG is a more economical blood glucose monitoring method and can achieve a similar performance in glycemic control as CGM, while CGM is beneficial for ideal GWG.

## 1 Introduction

Gestational diabetes mellitus (GDM) is one of the most common metabolic complications in pregnancy, and its global prevalence is as high as 14.0% (1). Gestational hyperglycemia increases the risk of maternal and fetal perinatal complications, which increases medical costs as well (2–4). Glycemic control can reduce perinatal complications, and blood glucose monitoring is a cornerstone of maintaining optimal blood glucose. Although hemoglobin A1c (HbA1c) levels fall during pregnancy due to physiological increases of red blood cell turnover, guidelines recommend an optimal target of HbA1c<6% during pregnancy without significant hypoglycemia (5). Additionally, as HbA1c represents an integrated measure of glucose, it may not fully capture postprandial hyperglycemia. Given the limitations of HbA1c assessment for blood glucose levels during pregnancy, patients need alternative blood glucose monitoring options.

Self-monitored blood glucose (SMBG) refers to monitoring fingertip blood glucose with a glucose meter, which represents an economical and convenient way of understanding real-time glucose readings (6). Continuous glucose monitoring (CGM) technologies assess dynamic glucose levels in daily life to measure the duration and magnitude of fluctuation, especially for fasting and postprandial measurements, for 72 h to 14 days (7). The American Diabetes Association (ADA) suggests that the use of CGM during pregnancy is associated with improved HbA1c levels and neonatal outcomes for pregnant women with type 1 diabetes (5). However, CGM use in GDM women is controversial. Several clinical trials have shown that the use of CGM during pregnancy in women with GDM was associated with better metabolic control and a reduced risk of macrosomia compared with women using SMBG only (8, 9). In contrast, some studies have found no significant differences in adverse pregnancy outcomes or glucose levels between CGM and SMBG users (10–12). Therefore, there is no consensus on CGM applications in GDM patients, especially the timing and frequency.

GDM is mild hyperglycemia in the second or third trimester, and studies have shown that approximately 80.6–91.2% of GDM cases have an HbA1c level < 6% (13, 14). Therefore, there is a need for prospective randomized clinical trials to clarify the possible benefit of using CGM in GDM women compared to only SMBG in those with milder GDM (HbA1c <6%) and to specifically clarify the CGM protocol during pregnancy. The purpose of the current study was to evaluate the effect of CGM technology on GDM compared with SMBG and to propose rational advances for glucose monitoring in mild GDM patients.

## 2 Materials and methods

The study was conducted at the endocrinology outpatient clinic of Shanghai General Hospital, Shanghai Jiao Tong University School of Medicine. The protocol was approved by the ethics committee of Shanghai General Hospital, Shanghai Jiao Tong University School of Medicine. Written informed consent was obtained from each patient. This trial was registered on ClinicalTrials.gov (NCT03955107).

### 2.1 Study participants

GDM women were diagnosed if one or more plasma glucose values during the 75-g oral glucose tolerance test (OGTT) at 24-28 gestational weeks met or exceeded the following values: 0 h, 5.1 mmol/L; 1 h, 10.0 mmol/L; and 2 h, 8.5 mmol/L (15). Major eligibility criteria included GDM patients with the following: aged 18–45 years old, 24-28 gestational weeks of pregnancy, singleton pregnancy, preconception body mass index (pre-BMI) ≥18 kg/m^2^, HbA1c<6% and voluntary completion of CGM. Exclusion criteria included pregestational type 1 diabetes mellitus (T1DM) or type 2 diabetes mellitus (T2DM).

### 2.2 Study design

**Figure 1:**
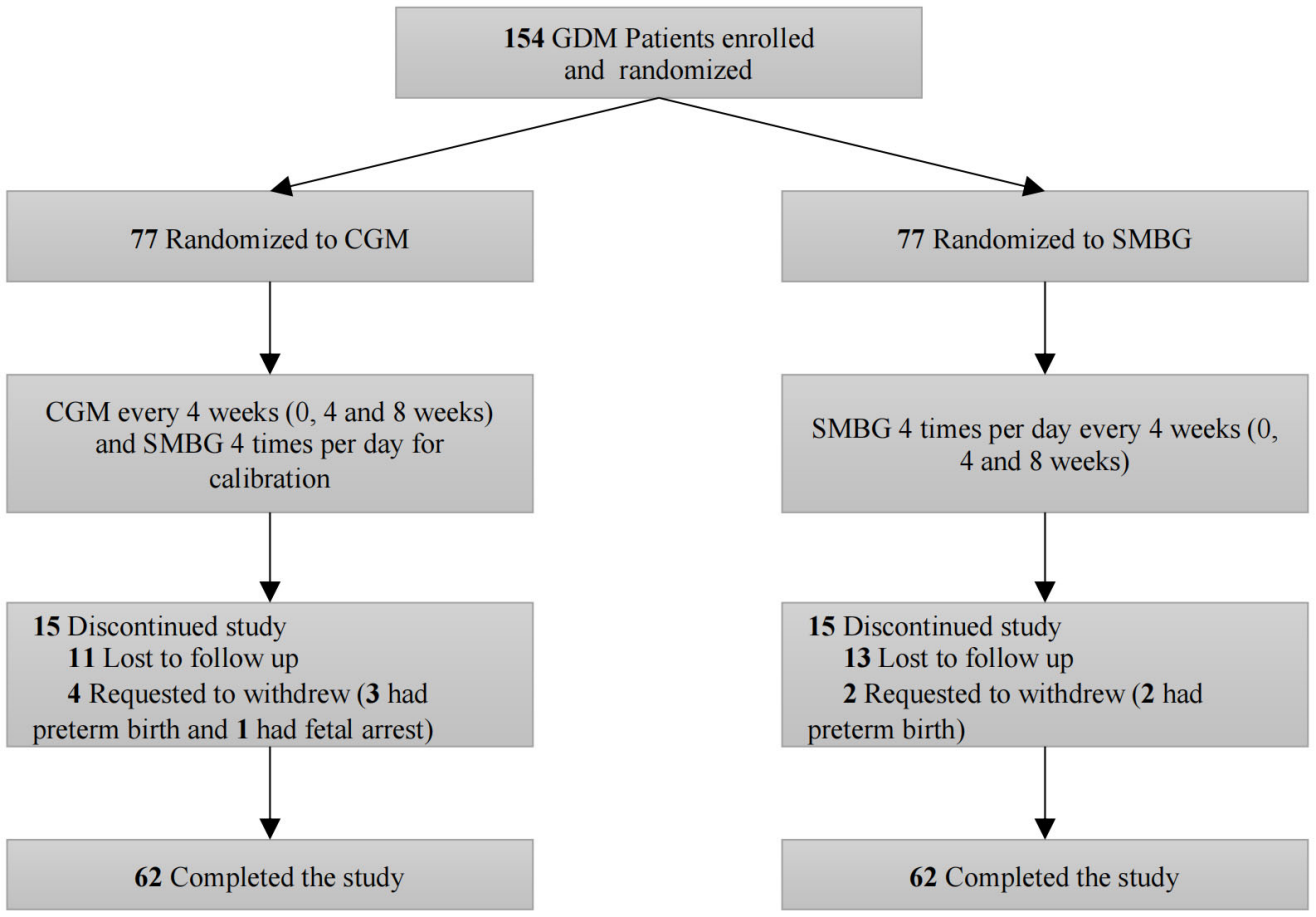
Study design. GDM, gestational diabetes mellitus; CGM, continuous glucose monitoring; SMBG, self-monitored blood glucose.

After verification of eligibility, each participant was assigned randomly to either the CGM in addition to SMBG or SMBG only group in a 1:1 ratio stratified by age (≤35 and >35 years old) at 24-28 gestational weeks. Study visits for both groups occurred 4 and 8 weeks after recruitment. Participants were provided with a CGM system (Medtronic Inc., Northridge, CA) that measured subcutaneous interstitial glucose for three consecutive days. The CGM system generated a daily record of 288 continuous sensor values. Capillary blood glucose readings should be measured at least four times per day by using a Freestyle Optium Neo (Abbott Diabetes Care Inc., USA) to calibrate the CGM system. The CGM group was instructed to use CGM every 4 weeks (0, 4 and 8 weeks) for a total of 3 times during the study. At the same time, the SMBG group was advised to perform SMBG 4 times per day for 3 consecutive days every 4 weeks (0, 4 and 8 weeks) and used additional CGM in blinded mode to collect glucose parameters for 3 days after 8 weeks. Participants in both groups continued their usual protocol of capillary glucose monitoring during their pregnancy and were asked to perform SMBG at least 7 times weekly (before meals, 2 h after meals and before bed) at other times (**Figure 1**). In addition, participants in both groups were provided with diabetes education, and clinicians reviewed CGM downloaded glucose data or SMBG data at each visit for treatment adjustment. The treatment goal was as follows: preprandial blood glucose level 3.3-5.3 mmol/L, 1 hour postprandial blood glucose (1h PG) 3.3-7.8 mmol/L and 2h PG 3.3-6.7 mmol/L (8). Insulin was offered if FBG > 5.3 mmol/L, 1 h PG > 7.8 mmol/L or 2 h PG > 6.7 at least twice according to the SMBG data. For patients in the CGM group, insulin treatment was determined by results of CGM when postprandial glucose levels rose above 7.8 mmol/L or FBG > 5.3 mmol/L for more than 10 minutes. Insulin dosages were adjusted according to glucose levels as the pregnancy continued.

### 2.3 Standard CGM metrics

After the 3-day blood glucose monitoring period, CGM metrics were calculated. Metrics for each patient in day 2 were used for quantification of glycemic variability (GV). The mean of daily differences (MODD) calculation is based on the data of 2 integrated consecutive days (day 2 and day 3). The time in range (TIR) was defined as the percentage of time within the target glucose range of 3.3–7.8 mmol/L during a 24-h period. The time above range (TAR) and time below range (TBR) were above the target glucose range of 7.8 mmol/L and below the target glucose range of 3.3 mmol/L, respectively. GV parameters included the standard deviation (SD) of sensor glucose values, the glucose coefficient of variation (CV), the large amplitude of glycemic excursions (LAGE), the mean amplitude of glycemic excursions (MAGE), MODD and the postprandial glucose excursion (PPGE) (16). The CV was calculated by dividing the SD by the mean of the corresponding glucose readings. LAGE was defined as the difference between the maximum and minimum glucose levels. MAGE was calculated by measuring the arithmetic mean of the differences between consecutive peaks and nadirs, and only excursions of more than one SD of the mean glycemic value were considered. MODD was the mean absolute value of the differences between glucose values during two successive 24-h periods. PPGE was defined as the difference between the highest postprandial glucose and fasting glucose levels.

### 2.4 Metabolic and clinical measurements

Each participant was administered a 75-g OGTT. In addition, serum total cholesterol (TC), triglyceride (TG), high-density lipoprotein-cholesterol (HDL-C), low-density lipoprotein-cholesterol (LDL-C), HbA1c and glycated albumin (GA), were detected. HOMA-β and HOMA-IR were calculated to evaluate β-cell function and insulin resistance using the following formulas: HOMA-β=20×FINS (μU/ml)/(FPG (mmol/L)-3.5) and HOMA-IR=FPG (mmol/L)×FINS (μU/mL)/22.5 (17). In addition, HbA1c and GA were detected before delivery. Gestational weight gain (GWG) was calculated by subtracting the weight before pregnancy from the weight before delivery. The GWG result was categorized into three groups, including below, within or above the recommended GWG for each prepregnancy BMI based on the Chinese Medical Association guidelines (2022). The Obstetrics and Gynecology Branch of the Chinese Medical Association has recommended weight gain values of 11.0–16.0 kg for underweight women (BMI <18.5 kg/m^2^), 8.0–14.0 kg for normal weight (18.5 ≤BMI <24.0 kg/m^2^), 7.0–11.0 kg for women who are moderately overweight (24.0 ≤BMI <28.0 kg/m^2^) and ≤9.0 kg for obese women (BMI ≥28.0 kg/m^2^).

### 2.5 Outcomes

The primary outcome was CGM-measured TIR at 8 weeks after enrollment. The secondary outcomes included HbA1c level before delivery; GWG; birth weight of the newborn; CGM-measured GV; insulin treatment; adverse pregnancy outcomes including gestational hypertension, preeclampsia, rate of cesarean, preterm delivery, macrosomia (birthweight ≥4 kg), large or small for gestational age (birthweight percentile >90th or <10th, LGA or SGA) and composite neonatal outcome (birth injury, neonatal hypoglycemia, hyperbilirubinemia, neonatal respiratory distress syndrome, and neonatal intensive care unit admission >24 h).

### 2.6 Cost of glucose monitoring during pregnancy

The two groups had the same cost for SMBG. The test strip cost was calculated assuming a cost of ¥3.6 per test strip and lancet. Each participant was distributed 33 strips and lancets every 4 weeks, for a total of 99 strips and lancets. The glucometer cost was estimated to be ¥396 for each participant. The additional cost for CGM was calculated to be ¥550 for sensor and ¥200 for receiver use for 3 days each visit, and totally three times in the study.

### 2.7 Statistical analysis

The study was powered to detect an increase in TIR from 94 ± 8% to 98 ± 8%. Considering a p 20% drop-out rate, we needed a total of 154 participants (77 in both groups) (alpha-error.025; beta-error.20; one-sided test).

Data were expressed as the mean ± standard deviation or the median with the interquartile range. Normally distributed continuous variables were compared by Student’s t test, while nonnormally distributed continuous variables were analyzed by the Mann−Whitney U test. The HbA1c and GA levels before delivery were analyzed according to the analysis of covariance method, adjusting baseline levels. Categorical variables were analyzed by the chi-square test, resulting in a relative risk (RR) with an accompanying 95% confidence interval (CI). Statistical significance was set at *p*<0.05. We used SPSS version 26 (IBM Corp., Armonk, NY) for statistical analyses.

## 3 Results

Between January 2019 and February 2021, 154 participants were randomly assigned to the CGM group (n=77) or SMBG group (n=77). The study outcome visit was completed by 62 participants (82.7%) in the CGM group and 62 (82.7%) in the SMBG group (**Figure 1**). Participant clinical characteristics were shown in **Table 1**. The participants in the SMBG group had higher fasting insulin (FINS) levels (57.10 (44.7-84.70) pmol/L versus 50.30 (39.90-73.15) pmol/L, p=0.036) and higher 1-h insulin (1hINS) levels (447.00 (268.80-668.30) pmol/L versus 330.35 (251.65-473.95) pmol/L, p=0.018), but there were no differences in HOMA-β, HOMA-IR or other metabolic characteristics between the two groups (**Table 1**).

**Table 1:** Demographic and metabolic characteristics of GDM patients at baseline.

	CGM (N=62)	SMBG (N=62)	*p*
**Age (years)**	31.81 ± 4.33	31.77 ± 3.77	0.965
**DM family history**	14 (22.6)	13 (21.7)	0.903
**Pre-BMI (kg/m^2^)**	22.23 ± 3.57	23.05 ± 3.53	0.202
**Chronic hypertension before pregnancy**	1 (1.6)	0 (0)	0.315
**Polycystic ovary syndrome**	4 (6.5)	7 (11.3)	0.343
**FPG (mmol/L)**	4.97 ± 0.52	4.92 ± 0.56	0.567
**1hPG (mmol/L)**	10.02 ± 1.66	10.15 ± 1.35	0.638
**2hPG (mmol/L)**	8.47 ± 1.66	8.61 ± 1.32	0.615
**FINS (pmol/L)**	50.30 (39.90-73.15)	57.10 (44.7-84.70)	0.036
**1hINS (pmol/L)**	330.35 (251.65-473.95)	447.00 (268.80-668.30)	0.018
**2hINS (pmol/L)**	359.45 (218.05-507.35)	434.60 (241.10-691.80)	0.059
**HOMA-β**	227.20 (126.95-390.62)	245.35 (121.94-350.15)	0.508
**HOMA-IR**	1.56 (1.19-2.44)	1.92 (1.27-2.71)	0.113
**HbA1c (%)**	4.94 ± 0.34	5.01 ± 0.36	0.248
**GA (%)**	13.31 ± 1.65	13.15 ± 1.76	0.620
**HbA1c <6%**	62 (100)	62 (100)	1.000
**TC (mmol/L)**	5.77 ± 1.00	5.77 ± 0.97	0.983
**TG (mmol/L)**	2.41 ± 0.95	2.79 ± 1.28	0.062
**HDL-C (mmol/L)**	1.87 ± 0.38	1.89 ± 0.47	0.722
**LDL-C (mmol/L)**	2.93 ± 0.91	2.82 ± 0.81	0.446

Data are expressed as mean ± SD or median (interquartile range) or n (%). pre-BMI, body mass index before pregnancy; FPG, fasting plasma glucose; 1hPG, 1-h plasma glucose; 2hPG, 2-h plasma glucose; FINS, fasting insulin; 1hINS, 1-h insulin; 2hINS, 2-h insulin; HbA1c, hemoglobin A1c; GA, glycated albumin; HOMA-IR, homeostasis model assessment for insulin resistance index; HOMA-β, homeostasis model assessment for β-cell function, TC, total cholesterol; TG, triglyceride; HDL-C, high-density lipoprotein-cholesterol; LDL-C, low-density lipoprotein-cholesterol.

### 3.1 CGM parameters evaluated by CGM after 8 weeks

The CGM metrics after 8 weeks were shown in **Table 2**. There were no differences in mean blood glucose (MBG) levels and glucose variability measures between the CGM and SMBG groups, and the TIRs were similar (100.00% (93.75-100.00%) in the CGM group and 99.14% (90.97-100.00%) in the SMBG group).

**Table 2:** Continuous glucose monitoring metrics after 8 weeks.

	CGM (N=62)	SMBG (N=62)	*p*
**MBG (mmol/L)**	5.34 ± 0.55	5.34 ± 0.70	0.959
**SD (mmol/L)**	0.82 ± 0.30	0.88 ± 0.41	0.346
**CV (%)**	15.51 ± 6.00	16.41 ± 6.95	0.441
**LAGE (mmol/L)**	3.57 ± 1.24	3.82 ± 1.64	0.331
**MAGE (mmol/L)**	1.78 ± 0.92	2.03 ± 0.99	0.147
**MODD (mmol/L)**	0.96 ± 0.47	1.06 ± 0.68	0.394
**TAR (>7.8 mmol/L) (%)**	0.00 (0.00-3.12)	0.00 (0.00-2.08)	0.241
**TIR (3.3-7.8 mmol/L) (%)**	100.00 (93.75-100.00)	99.14 (90.97-100.00)	0.183
**TBR (<3.3 mmol/L) (%)**	0.00 (0.00-0.00)	0.00 (0.00-1.39)	0.674
**PPGE of breakfast (mmol/L)**	2.31 ± 1.09	2.45 ± 1.26	0.519
**PPGE of lunch (mmol/L)**	1.69 ± 1.12	1.77 ± 1.18	0.699
**PPGE of dinner (mmol/L)**	1.62 ± 1.26	1.56 ± 0.96	0.746

Data are expressed as the mean ± SD or median (interquartile range). MBG, mean blood glucose; SD, standard deviation; CV, glucose coefficient of variation; LAGE, large amplitude of glycemic excursions; MAGE, mean amplitude of glycemic excursions; MODD, mean of daily differences; TAR, time above range; TIR, time in range; TBR, time below range; PPGE, postprandial glucose excursion.

### 3.2 Glucose levels, treatment and weight control antepartum

There were no between-group significant differences in antepartum HbA1c levels and GA levels. The insulin treatment percentage seemed to be higher in the CGM group but did not reach statistical significance (p=0.610). In the CGM group, 59.7% of cases achieved qualified weight gain at the end of pregnancy, compared to 40.3% of cases in the SMBG group. The results demonstrated that the proportion of the within GWG recommendation group was higher in the CGM group (p=0.046; **Table 3**). Although CGM may increase the rate of qualified weight gain, the glucose levels were similar in the two groups.

**Table 3:** Glucose levels, treatment and weight control antepartum.

	CGM (N=62)	SMBG (N=62)	*p*
**HbA1c (%)**	5.31 ± 0.06	5.35 ± 0.57	0.599
**GA (%)**	12.20 ± 0.20	12.45 ± 0.19	0.355
**Insulin treatment (%)**	10 (16.1)	8 (12.9)	0.610
**GWG Recommendations**			0.046
**Below**	9 (14.5)	8 (12.9)	
**Within**	37 (59.7)	25 (40.3)	
**Above**	16 (25.8)	29 (46.8)	

Data are expressed as mean ± SD or n (%). HbA1c, hemoglobin A1c; GA, glycated albumin; GWG, gestational weight gain.

### 3.3 Pregnancy outcomes

Pregnancy outcomes were shown in **Table 4**. The participants in the SMBG group had a higher birth weight of newborns (3291.56 ± 386.59 g versus 3123.79 ± 369.58 g, p=0.015), but there were no differences in other maternal and neonatal adverse outcomes between the CGM and SMBG groups.

**Table 4:** Pregnancy outcomes of GDM patients.

	CGM (N=62)	SMBG (N=62)	RR (95% CI)	*p*
Maternal outcomes				
**Cesarean section**	34 (54.8)	36 (58.1)	0.94 (0.65-1.34)	0.717
**Hypertensive disorders (Pregnancy-induced hypertension, or preeclampsia)**	5 (8.1)	2 (3.2)	1.80 (0.55-5.87)	0.436
Neonatal outcomes				
**Birth weight**	3123.79 ± 369.58	3291.56 ± 386.59	/	0.015
**Preterm births**	5 (8.1)	1 (1.6)	3.10 (0.51-18.72)	0.207
**Macrosomia**	1 (1.6)	1 (1.6)	1.00 (0.25-4.04)	1.000
**Large for gestational age (>90th percentile)**	5 (8.1)	5 (8.1)	1.00 (0.52-1.91)	1.000
**Small for gestational age (<10th percentile)**	4 (6.5)	2 (3.2)	1.53 (0.49-4.80)	0.680
**Birth injury**	0 (0)	1 (1.6)	/	1.000
**Hyperbilirubinemia**	7 (11.3)	10 (16.1)	0.83 (0.53-1.29)	0.433
**Neonatal hypoglycemia**	1 (1.6)	1 (1.6)	1.00 (0.25-4.04)	1.000
**Respiratory distress**	2 (3.2)	1 (1.6)	1.51 (0.30-7.57)	1.000
**Neonatal intensive care unit admission >24 h**	7 (11.3)	9 (14.5)	0.87 (0.54-1.40)	0.592
Composite neonatal outcome*	8 (12.9)	12 (19.4)	0.80 (0.53-1.21)	0.329

Data are expressed as mean ± SD or n (%). Composite neonatal outcome comprises birth injury, hyperbilirubinemia, neonatal hypoglycemia, respiratory distress, and neonatal intensive care unit admission >24 h.

### 3.4 Cost of glucose monitoring during pregnancy

The cost of glucose monitoring was shown in **Table 5**. The cost of SMBG in all GDM women was ¥752.4, while the additional cost for GDM women in the CGM group was ¥2250. The cost of the CGM group increased almost threefold compared with the SMBG group, which was primarily attributable to the CGM application.

**Table 5:** Cost of Glucose Monitoring during Pregnancy.

		CGM (N=62)		SMBG (N=62)	
	Unit Cost	Amount	Cost Per Patient	Amount	Cost Per Patient
**Glucometer**	396	1	396	1	396
**Test strips**	3.6	99	356.4	99	356.4
**CGM (sensor)**	550	3	1650	0	0
**CGM (receiver use)**	200	3	600	0	0
**Total cost**	/	/	3002.4	/	752.4

Protocol: The CGM group was instructed to use CGM every 4 weeks (0, 4 and 8 weeks) for a total of 3 times during the study. SMBG for calibration at least four times a day in CGM period. The SMBG group was also advised to perform SMBG 4 times per day for 3 consecutive days every 4 weeks (0, 4 and 8 weeks). Participants in both groups were asked to perform SMBG at least 7 times weekly at other weeks.

## 4 Discussion

In this study, we assessed the effect of CGM application compared with SMBG in GDM patients with HbA1c levels <6%. There was no difference in GV, HbA1c levels or perinatal adverse events between the use of CGM and SMBG. However, the CGM group showed better gestational weight control and a lower birth weight of newborns. Concerning the high costs and comparable outcomes in glucose control and pregnancy complications with CGM in GDM patients, SMBG is recommended for those mild GDM patients (HbA1c levels <6%). However, the long-term effect of appropriate weight gain during gestation *via* CGM remains to be studied.

GV appears to be associated with the development of diabetes complications and has a significant impact on quality of life (18). In our results, GV, HbA1c levels and pregnancy outcomes between CGM and SMBG in this trial of GDM were in accordance with some previous studies. Alfadhli et al. recruited 130 patients with GDM (62 SMBG and 68 CGM). Patients wore real-time CGM for 3–7 days once within 2 weeks of GDM diagnosis. HbA1c, fasting glucose, postprandial glucose levels at the end of the pregnancy and pregnancy outcomes were similar in both groups (11). Wei et al. randomly assigned 120 pregnant women with GDM to two groups (58 CGM and 62 SMBG). The patients were asked to wear CGM for 2–3 days during gestational weeks 24 to 28 (second trimester) or 28 to 36 (third trimester). The study found no significant differences in HbA1c levels or prenatal or obstetric outcomes, e.g., cesarean delivery rate, macrosomia or neonatal hypoglycemia, between the two groups (12). Kestila et al. enrolled 73 women with GDM (36 CGM for 2 days and 37 SMBG), and no differences were found in terms of preeclampsia, pregnancy-induced hypertension, or cesarean section rate between the two groups (10). In contrast, other studies have found some significant differences. Voormolen et al. randomized 108 women with GDM (54 CGM and 54 SMBG), and glycemic control was assessed by CGM for 5–7 days every 6 weeks in the CGM group. The CGM group had a significantly lower incidence of preeclampsia but no differences in fetal outcomes (9). Zhang et al. admitted 110 GDM patients (55 CGM and 55 SMBG). Patients in the CGM group wore real-time CGM devices for 14 days once and showed a lower incidence of hypoglycemia and higher blood glucose monitoring compliance, but the study did not collect pregnancy outcomes (19). Yu et al. recruited 340 women with GDM (150 CGM and 190 SMBG), and the results showed that the CGM group, which was performed for 3 days every week, had a lower incidence of preeclampsia, primary cesarean section, and premature delivery and better fetal outcomes than the SMBG group, including macrosomia, LGA, neonatal hypoglycemia, neonatal hyperbilirubinemia, and neonatal respiratory distress syndrome (8). These differences might be attributed to the study population and CGM frequency and duration. It seemed that patients with frequent CGM or wearing more time achieved better blood glucose management than SMBG. However, considering the cost, patient compliance and clinical practice, it is unrealistic to persuade GDM women to perform CGM frequently in the real world. For CGM frequency in GDM women, we suggest that monthly CGM is a feasible management plan. However, in our study, we found no significant difference between glycemic levels and adverse pregnancy outcomes between the two groups.

Meanwhile, CGM examination might improve patients’ consciousness of self-management for better weight control. Our results demonstrated that compared to the SMBG group, the proportion of the within GWG recommendation group was higher in the CGM group. In addition, our results favored a significant reduction in neonatal birthweight in the CGM group, but the proportions of macrosomia, LGA and SGA were not different. A study showed that excessive weight gain occurred in 31.8% of GDM women (20). GWG is related to neonatal birth weight, and excessive GWG increases the risk for macrosomia and LGA (21). Greater birth weight and weight gain in the first years of life have been found to be associated with increased BMI in adolescence (22). In addition, studies have shown a 33%-40% increased risk of overweight or obesity in children of mothers with excessive GWG (23, 24). Therefore, women with GDM should give more attention to GWG and infant birth weight, which can be improved by CGM examination in our study. However, whether this change in birth weight will affect the long-term health of the offspring remains to be studied.

Our study showed that insulin use in the CGM group was higher than that in the SMBG group, but the difference was not significant. Previous findings have shown that insulin use was more common in the CGM group than in the SMBG group, which demonstrated the advantages of CGM in the accurate detection of hyperglycemia and hypoglycemia (12). However, SMBG can achieve a similar performance compared with CGM for GDM patients who have mild dysglycemia. The total cost of diabetes care for China has been steadily rising, so it is important to evaluate the clinical application and economic value of new glucose-control technologies. Despite higher costs, for adults with T1D and suboptimal glycemic control, CGM is cost-effective with improved glucose control and reductions in nonsevere hypoglycemia compared to SMBG (25). The cost of CGM in T1D pregnancies was offset by improved maternal and neonatal outcomes (26). However, in GDM patients, our results showed no significant differences in glucose control or pregnancy outcomes between the CGM and SMBG groups. Overall, our results suggested that SMBG fits the need for an optimally effective glucose monitoring method at a low cost for GDM patients.

This study had some strengths and limitations. The strengths of the trial included the high adherence to group assignment and a protocol practical in clinical practice. Assignment could not be blinded because of the nature of the intervention, but the groups had a similar number of visits. This study focused on the effect and cost between CGM and SMBG in GDM patients with HbA1c<6%. The TIR and other metrics in the two groups reflected well-controlled blood glucose levels in all participants. However, our study also had several limitations. Although every effort was made to ensure complete datasets, this was not achieved because of missing or lost samples. The main reasons included premature delivery, referral to another hospital and the COVID-19 outbreak. However, the percentage of missing data (19.4%) fell within the prespecified power calculation assumptions. In addition, all the patients who enrolled had HbA1c levels lower than 6.0%; therefore, our results may not apply to individuals with GDM who have HbA1c levels higher than 6.0%. In addition, the long-term effects of maternal GWG and birth weight on children remain to be studied.

In conclusion, our data showed that for GDM patients with HbA1c less than 6%, regular SMBG is a more economical blood glucose monitoring method and can achieve similar performance as CGM in glycemic control and perinatal outcomes, while CGM is beneficial for ideal GWG.

## Data availability statement

The raw data supporting the conclusions of this article will be made available by the authors, without undue reservation.

## Ethics statement

The studies involving human participants were reviewed and approved by the ethics committee of Shanghai General Hospital, Shanghai Jiao Tong University School of Medicine. The patients/participants provided their written informed consent to participate in this study.

## Author contributions

ML and YW conceived of the design of the study and drafted the manuscript. JW, JY, YG, FF, NL and XX contributed to the data collection. MK participated in the data analysis. All authors contributed to the article and approved the submitted version.
